# Development of a High-Throughput Assay for Identifying Inhibitors of TBK1 and IKKε

**DOI:** 10.1371/journal.pone.0041494

**Published:** 2012-07-30

**Authors:** Jessica E. Hutti, Melissa A. Porter, Adam W. Cheely, Lewis C. Cantley, Xiaodong Wang, Dmitri Kireev, Albert S. Baldwin, William P. Janzen

**Affiliations:** 1 Lineberger Comprehensive Cancer Center, University of North Carolina (UNC) at Chapel Hill, Chapel Hill, North Carolina, United States of America; 2 Division of Medicinal Chemistry and Natural Products, Center for Integrated Chemical Biology and Drug Discovery, University of North Carolina at Chapel Hill, Chapel Hill, North Carolina, United States of America; 3 Division of Signal Transduction, Beth Israel Deaconess Medical Center, Boston, Massachusetts, United States of America; 4 Department of Systems Biology, Harvard Medical School, Boston, Massachusetts, United States of America; 5 Department of Biology, UNC at Chapel Hill, Chapel Hill, North Carolina, United States of America; Johns Hopkins School of Medicine, United States of America

## Abstract

IKKε and TBK1 are noncanonical IKK family members which regulate inflammatory signaling pathways and also play important roles in oncogenesis. However, few inhibitors of these kinases have been identified. While the substrate specificity of IKKε has recently been described, the substrate specificity of TBK1 is unknown, hindering the development of high-throughput screening technologies for inhibitor identification. Here, we describe the optimal substrate phosphorylation motif for TBK1, and show that it is identical to the phosphorylation motif previously described for IKKε. This information enabled the design of an optimal TBK1/IKKε substrate peptide amenable to high-throughput screening and we assayed a 6,006 compound library that included 4,727 kinase-focused compounds to discover *in vitro* inhibitors of TBK1 and IKKε. 227 compounds in this library inhibited TBK1 at a concentration of 10 µM, while 57 compounds inhibited IKKε. Together, these data describe a new high-throughput screening assay which will facilitate the discovery of small molecule TBK1/IKKε inhibitors possessing therapeutic potential for both inflammatory diseases and cancer.

## Introduction

The IKK family of kinases consists of four family members, the canonical IKKα and IKKβ, as well as two noncanonical family members, IKKε and TBK1. Together, this family of kinases regulates a myriad of critical cellular processes including inflammation, survival, proliferation, senescence, and autophagy [1]–[4]. Consistent with these numerous functions, aberrant IKK signaling can result in susceptibility to diseases such as inflammatory disorders and cancer [1], [3], [5], [6]. The canonical IKK complex, which consists of IKKα, IKKβ, and a regulatory subunit, NEMO, is a point of convergence for a variety of stimuli. Upon activation, the canonical IKKs, primarily IKKβ, phosphorylate IκBα, the inhibitor of NF-κB, which promotes the ubiquitination and degradation of IκBα [3], [7], [8]. The transcription factor NF-κB is then freed to accumulate in the nucleus and activate the transcription of a number of target genes involved in inflammatory and stress responses [3], [7], [8].

In contrast to the canonical IKKs, IKKε and TBK1 are activated by a smaller subset of inflammatory stimuli, and are especially critical for antiviral responses [6], [7], [9]. These kinases phosphorylate and activate the transcription factors IRF3, IRF7, and STAT1, promoting a Type 1 interferon response [10]–[14]. These kinases also activate NF-κB, but the mechanism by which this occurs in unclear since they do not phosphorylate both of the serines on IκBα which are required for IκBα degradation [15], [16]. IKKε and TBK1 can also promote oncogenesis. For example, IKKε is overexpressed in some breast and ovarian cancers, and TBK1 was recently shown to be important for Ras-induced cell transformation [17]–[20]. In spite of the important role these kinases play in both inflammatory and oncogenic signaling, few inhibitors have been identified. BX-795, a small molecule inhibitor of 3-phosphoinositide-dependent protein kinase 1 (PDK1), inhibits both IKKε and TBK1 at low nanomolar concentrations *in vitro* (IC_50_ at 41 nM and 6 nM, respectively) [21], [22]. However, BX-795 lacks selectivity as 16 out of 76 tested kinases were inhibited by BX-795 in the nM range [21]. It was also recently shown that a series of azabenzimidazole derivatives inhibits these kinases in the low nM range, but 6 of 79 kinases tested using one of these compounds were inhibited in a range within 10-fold of TBK [23]. These results suggest that IKKε and TBK1 are suitable targets for small molecule inhibitor development, but the need for the development of selective inhibitors of IKKε and TBK1 remains.

The development of high throughput assays to identify inhibitors of TBK1 and IKKε was hindered until recently by the absence of information regarding the substrate specificities of these enzymes. Peptide substrates for IKKε and TBK1 are frequently based on the IKKβ phosphorylation sites in IκBα, even though there is no evidence that all IKK family members phosphorylate the same substrate repertoires. In fact, the recently published phosphorylation motifs for IKKα, IKKβ and IKKε suggest that these kinases do have overlapping, but quite different, optimal peptide substrates, although a detailed comparison of the ability of IKK family members to phosphorylate these different peptide substrates has not been performed [24]–[26]. The phosphorylation motif for TBK1 has not been previously reported.

Here, a positional scanning peptide library (PSPL) technology was used to determine the optimal phosphorylation motif for TBK1. We demonstrate that the substrate specificity of TBK1 is identical to that of IKKε, but differs from the phosphorylation motif of IKKβ at key positions. Importantly, we also demonstrate that, like IKKε, TBK1 phosphorylates its predicted optimal peptide (TBK1-Tide) more efficiently than an optimal peptide for IKKβ or a peptide containing the IKKβ phosphorylation sites present in IκBα. We then used this information to develop and validate an IKKε/TBK1 peptide substrate appropriate for high-throughput chemical screening and executed a high-throughput screen (HTS) against both TBK and IKKε.

## Results

**Figure 1:**
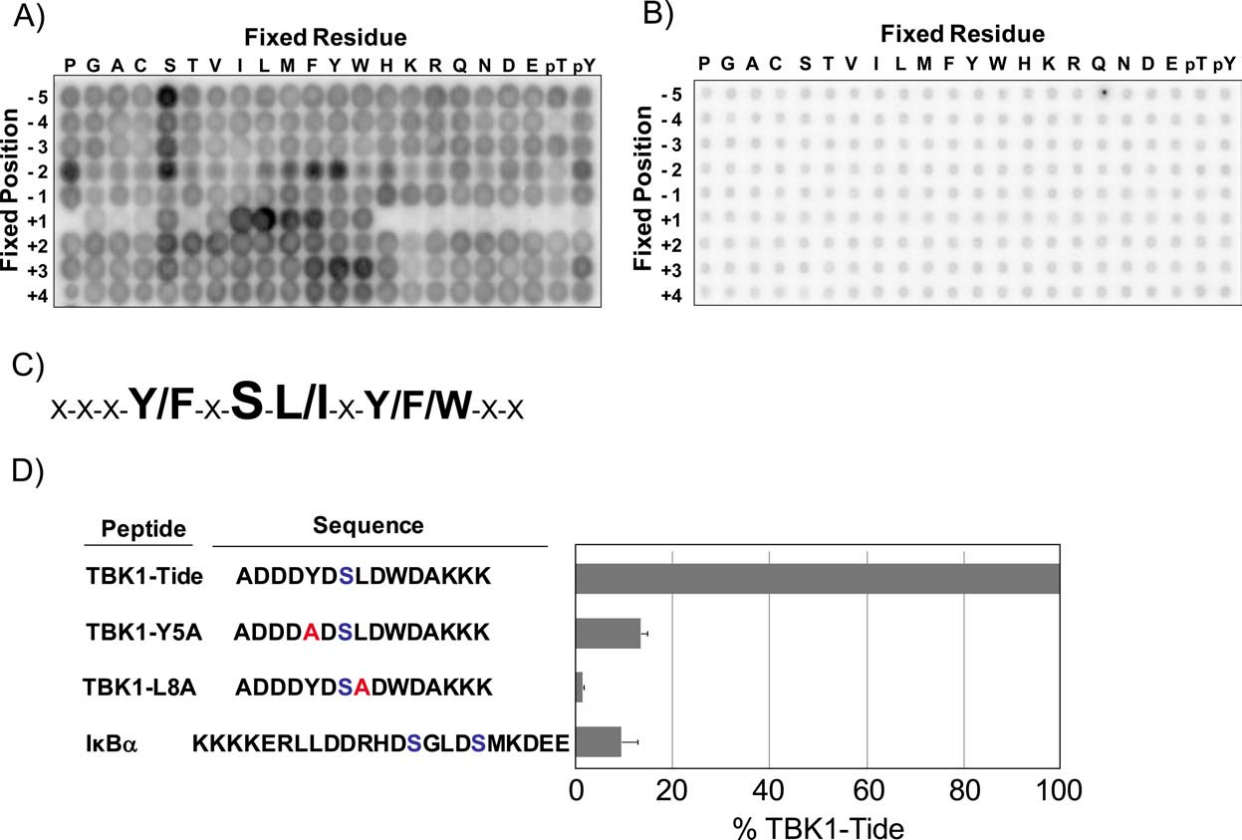
Identification of the optimal phosphorylation motif for TBK1. A-B) The positional scanning peptide library technology was used to determine the optimal phosphorylation motif for recombinant A) GST-TBK1 WT or B) kinase-dead GST-TBK1 K38A as described previously [27], [28]. Briefly, 198 peptide libraries were phosphorylated in individual kinase assays. The sequence for these libraries is Y-A-X-X-X-Z-X-S/T-X-X-X-X-A-G-K-K-biotin (Z =  fixed amino acid, X =  equimolar mixture of amino acids excluding Ser, Thr, and Cys). After binding to a streptavidin-coated membrane, phosphorylation was visualized by the incorporation of ^32^P. C) Primary and secondary selections for TBK1, as determined in A). D) 50 µM of the indicated peptide was phosphorylated in an *in vitro* kinase assay with recombinant GST-TBK1 for 30 min. Phosphorylation of each peptide is shown as a percentage of the rate of phosphorylation of TBK1-Tide, the optimal peptide substrate for TBK1. Error bars are standard deviation.

While it is clear that misregulation of TBK1 activity can promote inflammatory disorders and may play a role in oncogenesis, the role of TBK1 in these signaling pathways is poorly understood. Determining the substrate specificity of TBK1, therefore, would facilitate both the prediction of novel TBK1 substrates and the development of high-throughput assays to identify effective TBK1 inhibitors. To this end, we utilized PSPL technology to determine the optimal TBK1 phosphorylation motif using GST-TBK1 purified from HEK293T cells [27], [28]. This technology employs 198 biotinylated peptide libraries, which are used as substrates in individual solution-based kinase assays. Each peptide library has a mixture of serine and threonine at a fixed central position and also has one other position fixed to one of the 20 naturally-occurring amino acids. Phosphothreonine and phosphotyrosine were also included at the fixed positions to allow the identification of primed phosphorylation events. All other positions contain a degenerate mixture of amino acids. Following a kinase reaction, the biotinylated peptides are captured with an avidin membrane and preferences for individual amino acids at each position can be examined via the incorporation of radiolabeled phosphate. This PSPL assay revealed that TBK1 has preferences at a number of positions relative to the phosphorylation site, while a kinase-dead GST-TBK1 K38A does not (Figures 1A-B). TBK1 has an absolute requirement for a hydrophobic residue at the +1 position relative to the phosphorylation site (Figures 1A, 1C, and S1). TBK1 also displays a strong preference for phenylalanine or tyrosine at the -2 position, and a minor preference for bulky hydrophobic residues at the +3 position (Figures 1A, 1C, and Figure S1). To confirm this phosphorylation motif, an optimal peptide (TBK1-Tide) was generated and was efficiently phosphorylated by TBK1 *in vitro*. In contrast, peptides in which the +1 leucine or -2 tyrosine are changed to alanine were no longer efficiently phosphorylated by TBK1 (Figure 1D).

TBK1 is highly homologous to the related kinase IKKε, and also shares significant homology with the canonical IKK family member IKKβ. The substrate specificities of IKKε, IKKα, and IKKβ have also recently been determined using the PSPL technology [24]–[26]. Not surprisingly, the phosphorylation motif for TBK1 is identical to that of IKKε. Interestingly, while both the noncanonical and canonical IKKs display preferences for hydrophobic residues at the +1 position and aromatic residues at the -2 position, the optimal phosphorylation motifs for these kinases differ at other positions. For example, while TBK1 prefers large aliphatic residues at the +3 position, IKKα and IKKβ prefer acidic residues at +3. In addition, the canonical IKKs display a strong preference for phosphorylated residues at the -4 and -5 positions, suggesting that these kinases can be primed by upstream phosphorylation events. However, no evidence of priming phosphorylation is observed for TBK1. Consistent with these data, a peptide substrate corresponding to the well-established IKKα/β phosphorylation sites on IκBα was phosphorylated by TBK1 much less efficiently than TBK1-Tide (Figure 1D).

**Figure 2:**
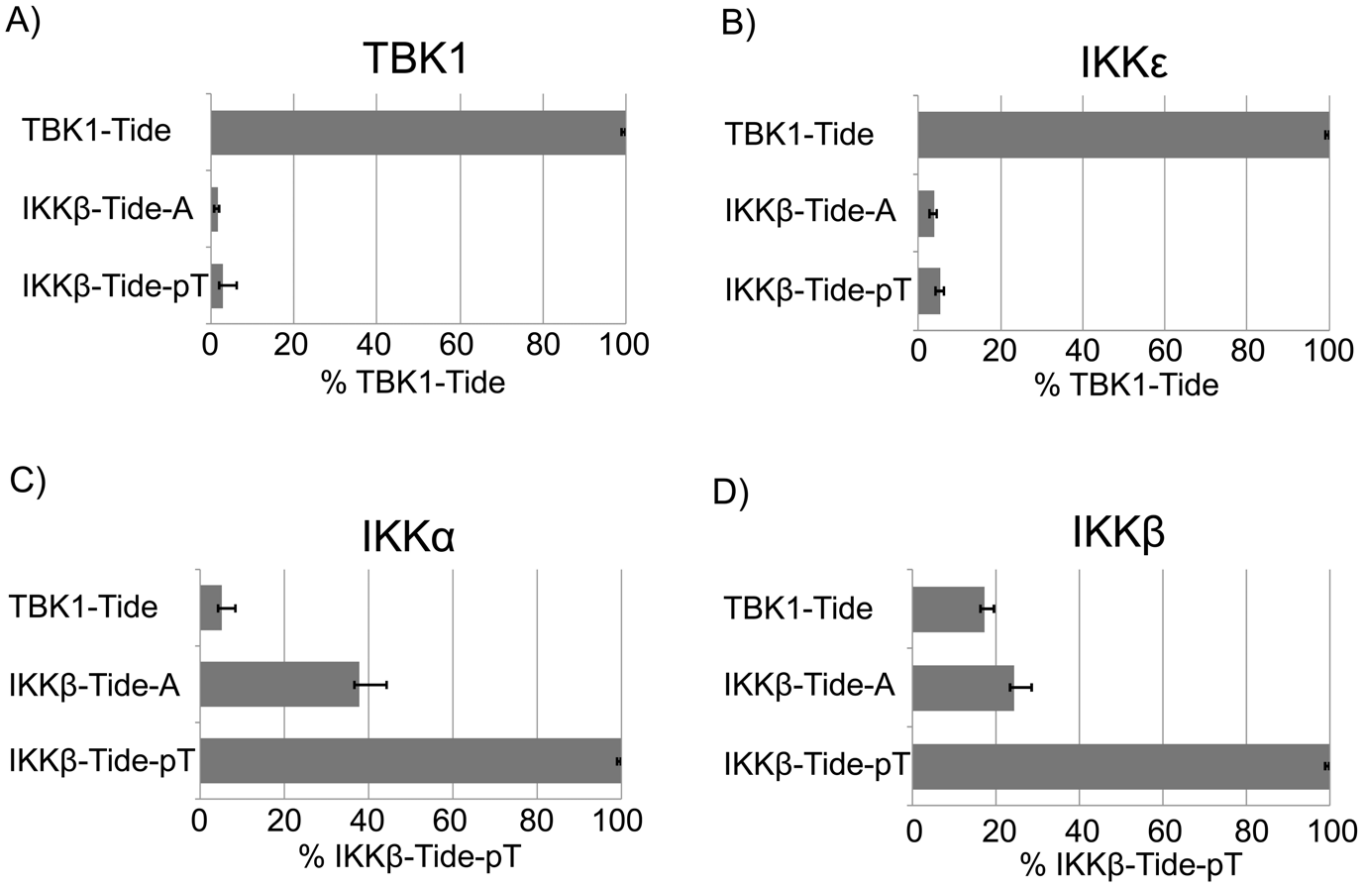
Members of the IKK family have different optimal peptide substrates. Recombinant GST-IKKα, GST-IKKβ, GST-IKKε, and GST-TBK1 were overexpressed in and purified from HEK293T cells. Each IKK family member was incubated in an *in vitro* kinase assay with 50 µM TBK1-Tide, IKKβ-Tide-pT, or IKKβ-Tide-A at 30°C for 45 min. TBK1-Tide  =  ADADYASLDWDAKK. IKKβ-Tide-pT  =  ADpTRYESIDEEAKKK. IKKβ-Tide-A  =  ADARYESIDEEAKKK. Phosphorylation of each peptide was measured for A) TBK1 B) IKKε C) IKKα and D) IKKβ. Error bars are standard deviation.

As the PSPL assays employ degenerate peptide mixtures, it was important to confirm differences in the substrate specificities among the IKKs using individual peptide substrates. To this end, the predicted optimal IKKβ substrate peptide (IKKβ-Tide-pT) was generated [25]. This peptide contains the +1 leucine and -2 tyrosine which are preferred by all IKK family members, but differs from TBK1-Tide at secondary positions. Importantly, this peptide contains a phosphothreonine residue at -4. We also generated a similar peptide (IKKβ-Tide-A) which is identical to IKKβ-Tide-pT except that it contains an alanine at the -4 position. All four IKK family members were then examined for their ability to phosphorylate TBK1-Tide, IKKβ-Tide-pT, and IKKβ-Tide-A. Indeed, Figures 2A-B show that TBK1 and IKKε strongly prefer to phosphorylate their optimal peptide, TBK1-Tide. Importantly, they also show no significant preference for IKKβ-Tide-pT over IKKβ-Tide-A, confirming that these kinases cannot be primed by upstream phosphorylation events. In contrast, IKKα and IKKβ strongly prefer to phosphorylate the optimal IKKβ substrate peptide, IKKβ-Tide-pT, over either TBK1-Tide or IKKβ-Tide-A, demonstrating their preference for the optimal IKKα/β substrate peptide and their ability to be primed by upstream phosphorylation events (Figures 2C-D). These data clearly show the importance of secondary and tertiary selections for the IKKs to properly identify their substrates. In addition, these data suggest that while TBK1 and IKKε may share a significant number of substrates, the canonical and noncanonical IKKs are likely to have somewhat overlapping, yet distinct, substrate pools.

**Figure 3:**
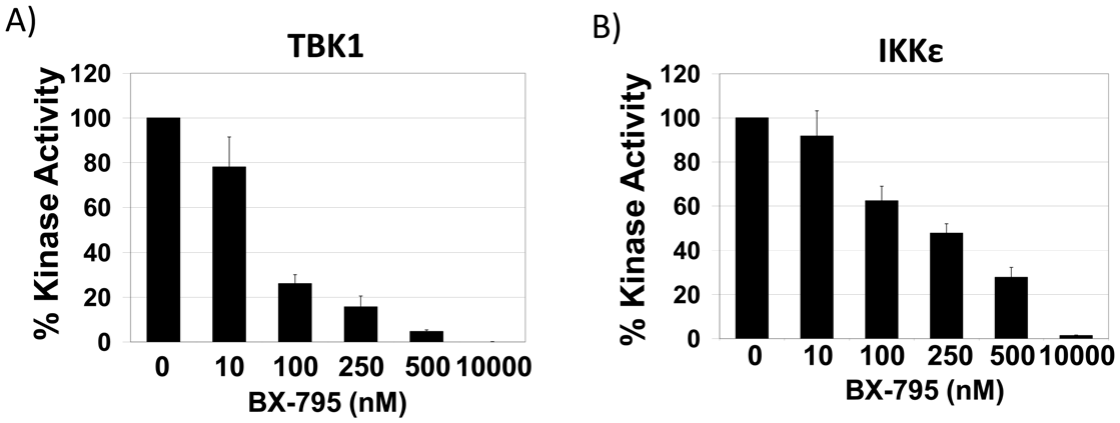
Inhibitor efficacy can be measured via phosphorylation of TBK1-Tide. A-B) Mixtures of A) TBK1 or B) IKKε with 50 µM TBK1-Tide were incubated with increasing concentrations of BX-795 for 20 minutes at RT. *In vitro* kinase reactions were then initiated with addition of γ^32^-P-ATP to a final concentration of 100 µM, and incubated at 30°C for 30 minutes. Error bars are standard deviation.

As few small molecule inhibitors of IKK family members with clinical potential have been identified, the development of effective screening technologies to identify novel inhibitors of IKK family members is of great interest. To validate that phosphorylation of TBK1-Tide can be blocked by a known TBK1/IKKε inhibitor, purified GST-TBK1 or GST-IKKε was incubated with 50 µM TBK1-Tide and increasing concentrations of a known TBK1/IKKε inhibitor, BX-795, for 20 minutes. An *in vitro* kinase reaction was then initiated by addition of γ^32^P-ATP, and incorporation of radiolabeled ATP was measured (Figure 3A-B). Indeed, phosphorylation of TBK1-Tide provides an effective read-out for the measurement of TBK1 and IKKε activity (Figure 3A-B).

**Figure 4:**
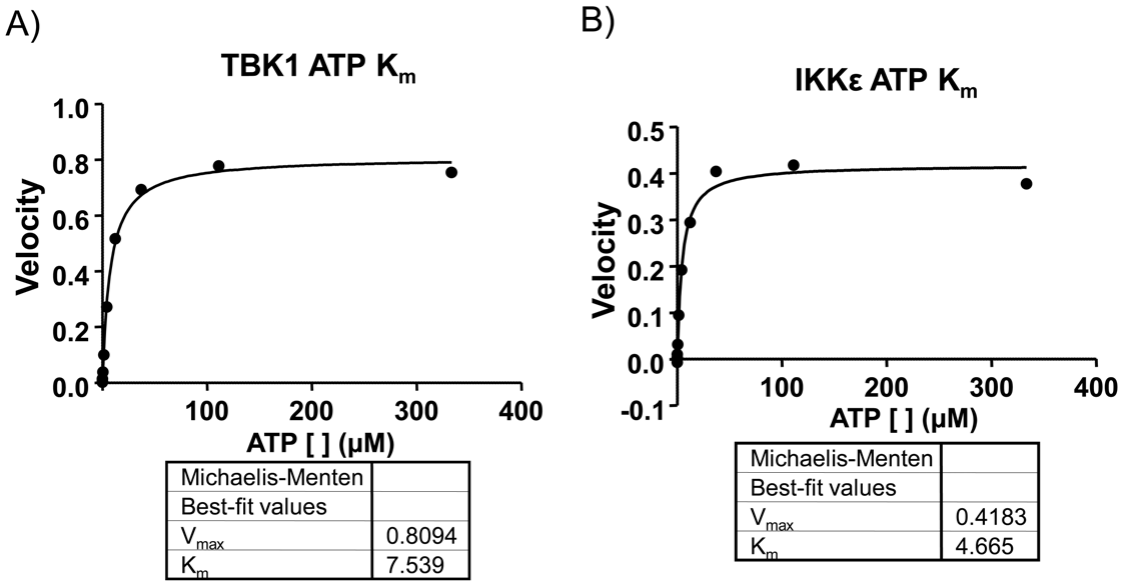
ATP K_m_ determination for TBK1 and IKKε. Enzymatic reactions of A) TBK1 or B) IKKε were incubated at room temperature with 10 ATP concentrations varying from 333 µM to 0.017 µM in three fold dilutions. Reactions were sampled on the Caliper EZReader system at 9.35 minute intervals over a 3 hour period. Percent conversions were calculated from relative heights of product and substrate peaks and used to calculate velocity and ATP K_m_ in Graph Pad Prism.

This validated substrate specificity was then used to develop assays for TBK1 and IKKε which are compatible with HTS using microfluidic capillary electrophoresis (MCE). MCE operates on the principle that small fluorescently-labeled peptide substrates from ∼100 nanoliter sized aliquots of reaction samples are separable in a capillary channel etched in a quartz microfluidic chip ratio when a current is applied [29], [30]. This technique has been widely adopted as a gold standard assay in the profiling of small molecule inhibitors of kinases and can be tested in a high-throughput fashion [31], [32]. TBK1-Tide was synthesized with an N-terminal 5– Carboxyfluorescein (5-FAM) dye for use as a substrate. The TBK1-Tide synthesized for HTS (5-FAM-Aha-ADADYASLDWDA-NH2) retained all of the residues critical for phosphorylation by TBK1 and IKKε, but had the -1 and -4 Asp residues changed to Ala in order to decrease the likelihood of Asp isomerization. TBK1 and IKKε were characterized for their behavior with this substrate and found to have K_m_ values for ATP of 7.5 µM and 4.7 µM respectively (Figure 4A-B). Because of the MCE system’s limits of detection, TBK1-Tide was fixed at a concentration of 1 µM and K_m_ values for substrate were not determined. The enzyme concentrations were then titrated and fixed to 120 nM for TBK1 and 81 nM for IKKε. These values were chosen to give 30% conversion of substrate to product after 2 hours of incubation (data not shown).

**Figure 5:**
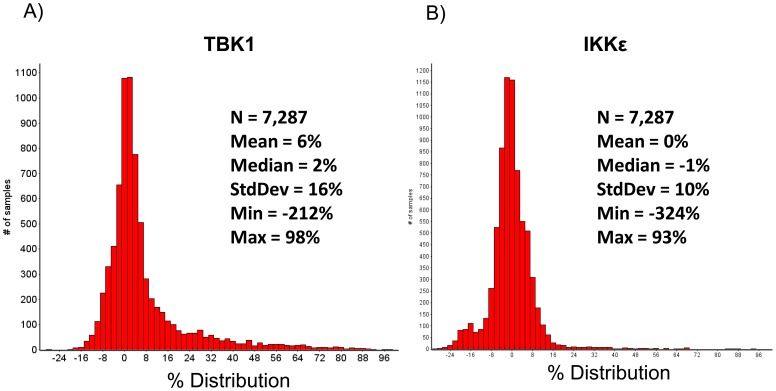
Distribution of compound activity. A–B) The kinase library was screened at 10 µM in a single concentration format against enzymatic reactions of A) TBK1 and B) IKKε. The distribution of activity is shown as a frequency histogram based on the number of compounds active at each level (% Inhibition). The data follow a normal distribution.

Compounds were screened at a final concentration of 10 µM in 0.1% DMSO. Two libraries of compounds were tested. The Library of Pharmaceutically Active Compounds (LOPAC) consists of 1280 known bioactive small molecules, including 300 FDA approved drugs including antibiotics, and compounds targeting gene regulation and expression, multi-drug resistance, apoptosis, ion channels, neurotransmission, calcium signaling, lipid signaling and phosphorylation regulation. This library was tested in duplicate to establish the reproducibility of the screen (Figure S2). A second kinase-focused library consisting of 4,727 unique and “rule of five” compliant compounds was also tested (Figures 5A and B) [33].

**Figure 6:**
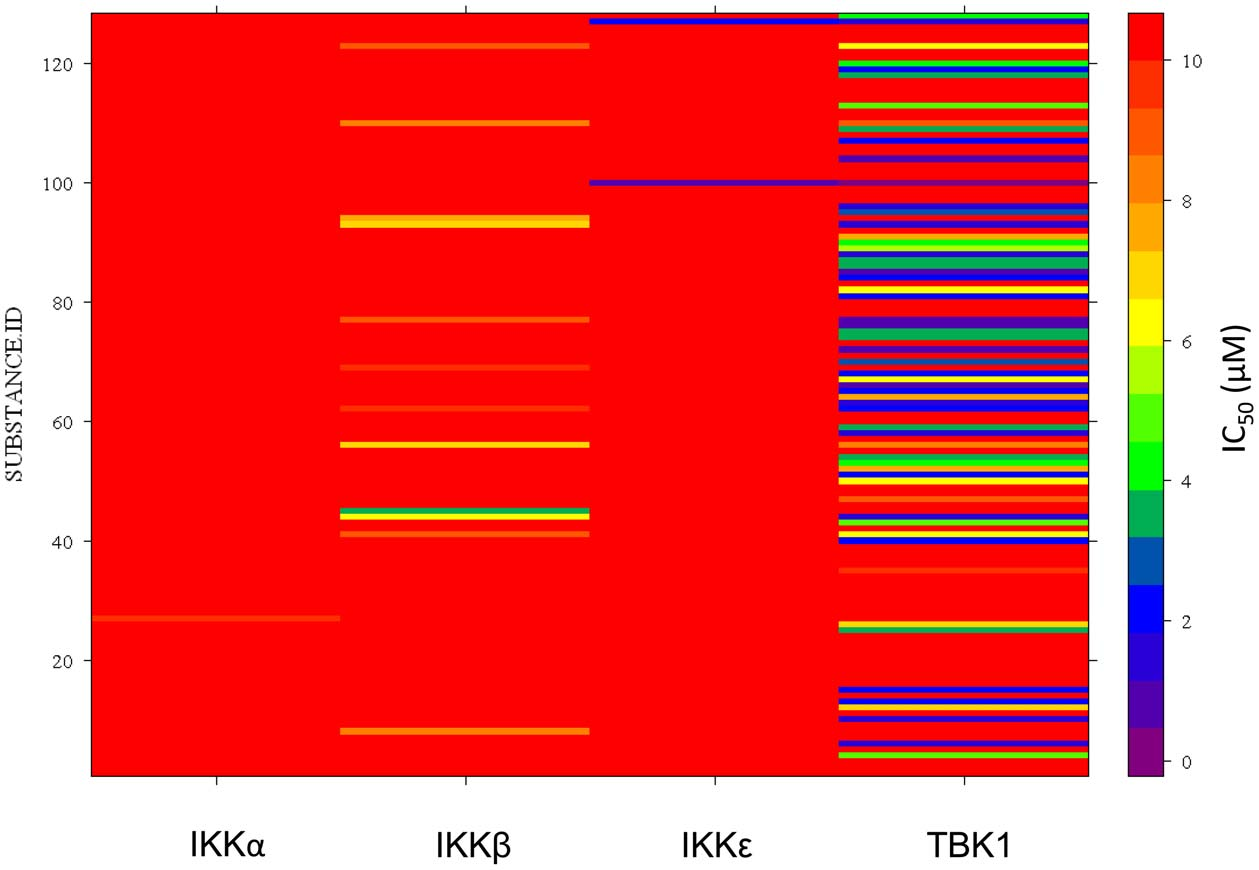
Relative activity of active compounds. Activity of selected compounds tested in the potency screen are shown as a heat map scaled from <1 µM (purple) to >10 µM (red) against IKKα, IKKβ, IKKε, and TBK1.

**Figure 7:**
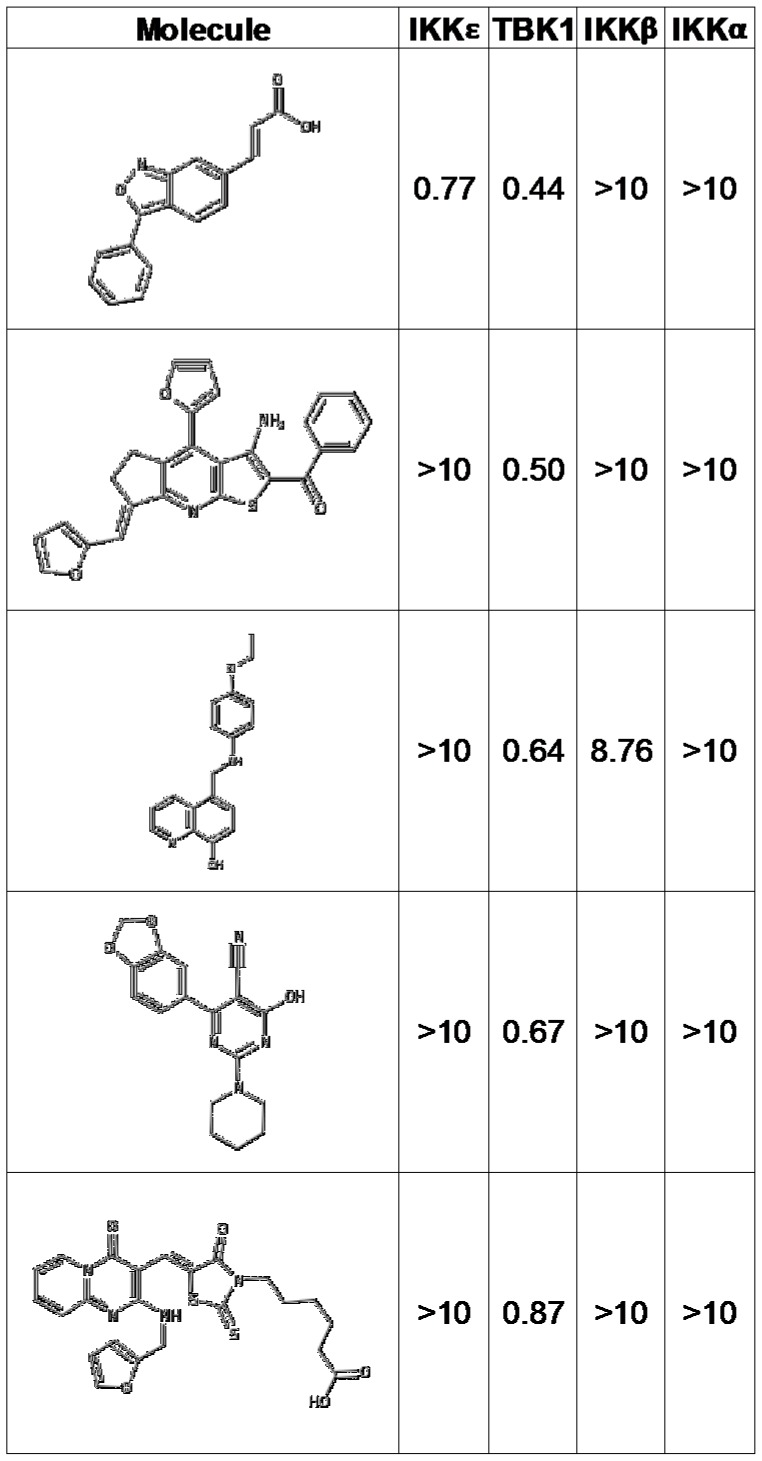
Compounds showing the most efficient inhibition of TBK1. IC_50_ values of the five most effective TBK1 inhibitors against each IKK family member are indicated (µM).

Results from the HTS were found to follow a quasi-normal distribution (Figures 5A and B) [34]. Compounds that showed inhibition values greater than three standard deviations from the mean value (50% inhibition for TBK1 and 30% for IKKε) were considered active. Active compounds from each target were clustered based on structural similarity using Pipeline Pilot software [35] and filtered for drug-like properties using the REOS filter (Table 1) [36]. Active compounds from the kinase cassette that showed selectivity for either enzyme were re-tested in 10 point dose response curves to establish potency values. This screen demonstrated that 227 compounds in the library inhibited TBK1 at a concentration of 10 µM and 57 compounds inhibited IKKε at a concentration of 10 µM (Figure 6). The structures of the five most active compounds are shown in Figure 7, including 4 TBK1-specific inhibitors and 1 dual TBK1/IKKε inhibitor. All of these compounds inhibited these kinases at concentrations of less than 1 µM.

**Table 1:** Activity comparison for TBK1 and IKKε.

TBK1	IKKε
N_unfiltered hits_ = 227 (@ inh.>50%)	N_unfiltered hits_ = 57 (@ inh.>30%)
N_filtered.drug-like_ = 184	N_filtered.drug-like_ = 33
N_LOPAC_ = 18	N_LOPAC_ = 12
N_Kinase lib_ = 166	N_Kinase lib_ = 21
N_clusters_ = 11 (N_in cluster_ ≥3)	N_clusters_ = 0 (N_in cluster_ ≥3)
N_singletons_ = 105	N_singletons_ = 27

Number of active compounds (N) detected in each screen for total number detected (unfiltered), number after drug like filtering (filtered.drug-like), hits from the LOPAC and Kinase libraries, and the number of chemical clusters and singleton hits as described in the text.

As IKKβ and IKKα are closely related to TBK1 and IKKε, it was important to determine the specificity of any candidate inhibitors. A secondary screen was therefore performed to evaluate the ability of the top-scoring TBK1 and IKKε inhibitors to inhibit the canonical IKKs. It was determined that IKKβ phosphorylated TBK1-Tide efficiently enough to perform this secondary screen, though for IKKα the commercially available Caliper FL-1 peptide was more efficiently phosphorylated than TBK1-Tide (data not shown). The ATP K_m_ of IKKβ for TBK1-Tide was 2 µM and the ATP K_m_ of IKKα for FL-1 was 134 µM (Figure S3). Enzyme concentrations were titrated and fixed to 11.5 µM for IKKα and 1.1 µM for IKKβ and the screen was performed as described above for TBK1 and IKKε. Importantly, few of the compounds which inhibited TBK1 or IKKε also inhibited IKKα or IKKβ, and none of the most effective TBK1/IKKε inhibitors were effective against IKKα and IKKβ (Figures 6, 7 and Table S1). Together, these data confirm that the inhibitors identified in this screen are specific for TBK1 or TBK1/IKKε among the IKK family members.

## Discussion

The development of effective small-molecule screening technologies for kinases is dependent on appropriately measuring changes in enzyme activity. While phosphorylation of a known protein substrate can be measured as a reporter for kinase activity, a peptide substrate is usually superior, as it is easier to generate large, consistent quantities, and is more amenable to the development of non-radioactive assays. However, the generation of an optimal peptide substrate requires a thorough understanding of kinase substrate specificity, and this information is only available for a small fraction of the >500 protein kinases in the human genome. The substrate specificities of three IKK family members, IKKα, IKKβ and IKKε, have recently been described [24]–[26].

Like IKKε, TBK1 is a noncanonical IKK family member which regulates Type I interferon signaling and may play a role in oncogenesis. Here, a positional scanning peptide library technology was utilized to identify the optimal phosphorylation motif for TBK1 (Figure 1) [27]. The substrate specificity of TBK1 is identical to that of related kinase IKKε. Interestingly, the substrate specificities of the noncanonical IKKs share overlapping characteristics with the substrate specificity of the canonical IKKs, but the optimal peptide substrates for these kinases are quite different (Figure 2). These data allowed the generation of a peptide substrate for TBK1 and IKKε (TBK1-Tide) which is amenable to high-throughput screening. This technology was then used to screen the LOPAC library and a kinase-focused library to discover *in vitro* inhibitors of TBK1 and IKKε. This HTS revealed that 227 compounds in this library inhibited TBK1 at a concentration of 10 µM and 57 compounds inhibited IKKε, including several compounds that inhibited these enzymes at sub-micromolar concentrations (Figure 7). Of the compounds tested in this screen, the molecules in the LOPAC library were of particular interest since this library contains known bioactive molecules. The best TBK1/IKKε inhibitors from the LOPAC library are therefore shown in Table S1. Unfortuntately, none of the compounds from the LOPAC library were among the best inhibitors of IKKε or TBK1, and many lacked specificity as they also inhibited IKKα (Table S1). Studies examining the ability of the compounds in Figure 7 to inhibit TBK1 or IKKε in cell-based assays are ongoing. As TBK1 and IKKε are points of convergence for both inflammatory and oncogenic signaling pathways, the further refinement of novel TBK1/IKKε inhibitors may provide powerful new therapeutic drugs for inflammatory disorders or cancer.

## Materials and Methods

### Antibodies, Plasmids, and Reagents

GST-TBK1 was created by PCR cloning into the BamHI site of the pEBG vector. GST-TBK1 K38A was created using a modification of the QuickChange Site-directed mutagenesis protocol (Stratagene). GST-IKKε, GST-IKKα, and GST-IKKβ for *in vitro* kinase assays were generated and used as previously reported [24]–[26]. TBK1-Tide (ADDDYDSLDWDAKKK), TBK1-Y5A (ADDDADSLDWDAKKK), and TBK1-L8A (ADDDYDSADWDAKKK) used in Figure 1 were created and HPLC purified under contract by the Tufts University Core Facility. The TBK1 peptide used in Figures 2 and 3 (ADADYASLDWDAKK) was generated to decrease the likelihood of Asp isomerization observed with the original TBK1-Tide. This peptide, as well as IKKβ-Tide-pT (ADpTRYESIDEEAKKK) and IKKβ-Tide-A (ADARYESIDEEAKKK) were also generated and HPLC purified by the Tufts University Core Facility. The IκBα (IKK substrate) peptide was obtained from Upstate. The FL-1 peptide (5-FAM-AKRRRLSSLRA-COOH) was obtained from Caliper Life Sciences. For all library screening, recombinant IKKα, IKKβ, IKKε and TBK1 were purchased from Life Technologies (Invitrogen).

### Cell Culture, Transfection, Immunoprecipitations, and Western Blotting

HEK-293T cells were obtained from ATCC and were grown in DMEM containing 10% FBS. Transfection was performed by polyethylenimine. For preparation of recombinant kinases, cells were lysed in 50 mM Tris (pH 7.5), 150 mM NaCl, 1% Triton X-100, 1 mM EDTA, 1 mM EGTA, 1 mM β-glycerophosphate, 1 mM PMSF, 1 mM sodium orthovanadate, 1 µg/mL leupeptin, 1 µg/mL pepstatin, and 10 nM Calyculin A.

### Design of 4,727 Member Kinase-focused Library

More than 100K compounds were initially reviewed in the form of SD files from Life Chemicals, ChemDiv, Asinex and Enamine. These kinase-focused libraries were designed by their respective vendors using one or more of the following approaches: 1) searching virtual and physical general purpose libraries for compounds similar to known kinase inhibitors, 2) selecting or synthesizing compounds having a hinge-binding motif, e.g. heterocycles with a high likelihood to bind the kinase hinge motif conserved in nearly every kinase-small molecule X-ray structure, and 3) structure- or ligand-based virtual screening on representative kinase structures. Following an analysis of each vendor’s library, the UNC CICBDD acquired 4,727 compounds (from all four vendors) that all were unique and “rule of five” compliant [33].

### High-throughput Screen for TBK1, IKKε, IKKα and IKKβ Inhibitors

5-FAM-labeled TBK1-Tide for library screening was generated in the High-Throughput Peptide Synthesis and Arrays Core Facility at University of North Carolina at Chapel Hill. The 1,280 compound Library of Pharmaceutically Active Compounds (Sigma) and the UNC CICBDD 4,727 compound kinase-focused library were evaluated for their ability to inhibit phosphorylation of 5-FAM-TBK1-Tide (TBK1, IKKε, IKKβ) or FL-1 (IKKα) using the MCE EZreader system from PerkinElmer.

### *In vitro* Kinase Assays

Kinase buffer contained 50 mM Tris (pH 7.5), 12 mM MgCl_2_, 1 mM β-glycerophosphate, 100 µM ATP, and 10 µCi γ-^32^P-ATP/reaction. Reactions were incubated at 30°C for 1h. Recombinant GST-TBK1 and GST-TBK1 K38A used in the positional scanning peptide library assay were generated as described above and the assay was performed as described previously [27], [28]. MCE assay buffer contained 50 mM Hepes (pH 7.4), 0.01% Triton X-100, 10 mM MgCl_2_, 1 mM DTT, 0.01% BSA and ATP at the determined K_m_ (4.7 µM for IKKε, 7.5 µM for TBK1, 134 µM for IKKα, and 2 µM for IKKβ). IKKε reactions were incubated for 2 hours at room temperature and TBK1, IKKα, and IKKβ reactions were incubated for 6 hours at room temperature.

## Supporting Information

Figure S1 **Selectivity values for TBK1.** Following the PSPL assay, relative affinities for each amino acid at each position relative to the phosphorylation site were calculated.
(PDF)

Figure S2 **Comparison of duplicate compound assay values.** A–B) The LOPAC library was screened in duplicate at 10 µM in a single concentration format against enzymatic reactions of A) TBK1 and B) IKKε. Results from the first determination are shown on the horizontal axis (Result 1) and the second determination is shown on the vertical axis (Result 2).
(PDF)

Figure S3 **ATP K_m_ determination for IKKα and IKKβ.** Enzymatic reactions of A) IKKα and B) IKKβ were incubated at room temperature with 10 ATP concentrations varying from 333 µM to 0.017 µM in three fold dilutions. Reactions were sampled on the Caliper EZReader system at 9.35 minute intervals over a 3 hour period. Percent conversions were calculated from relative heights of product and substrate peaks and used to calculate velocity and ATP K_m_ in Graph Pad Prism.
(PDF)

Table S1 **Most active compounds from the LOPAC set.** Values represent percent inhibition of the listed kinase isoform when treated with the indicated inhibitor at a concentration of 10 µM after 2 hours (at completion of the assay as described in the text).
(XLSX)
