# Supplementary material for: Development of a High-Throughput Assay for Identifying Inhibitors of TBK1 and IKKε
Source: PLoS One. 2012 Jul 30;7(7):e41494. doi: 10.1371/journal.pone.0041494 (PMC3408500; doi:10.1371/journal.pone.0041494)
Supplement: Figure S1 — Selectivity values for TBK1. Following the PSPL assay, relative affinities for each amino acid at each position relative to the phosphorylation site were calculated. (PDF) [file pone.0041494.s001.pdf]

Hutti, et al. Figure S1

	-5	-4	-3	-2	-1	+1	+2	+3	+4
P	0.96	1.15	1.16	1.99	1.29	0.08	0.63	0.80	0.50
G	0.92	1.06	1.42	0.54	0.56	0.50	0.88	0.72	0.62
A	0.97	0.61	0.70	0.52	0.82	0.25	0.83	0.59	0.86
C	0.72	0.78	0.46	0.51	0.58	0.36	0.63	0.74	0.77
S	3.33	2.22	3.00	2.49	1.06	1.46	1.63	1.43	0.79
T	1.10	1.18	1.00	0.73	1.11	0.49	1.43	0.76	0.98
V	0.78	0.66	0.41	0.55	0.71	1.14	1.40	0.77	1.16
I	0.73	0.84	0.24	0.31	0.70	2.43	1.35	1.08	1.22
L	0.82	0.85	0.65	0.97	0.98	3.72	1.22	1.19	1.26
M	0.61	0.91	0.99	1.39	1.51	2.80	1.39	1.06	1.15
F	0.87	0.92	0.78	2.12	1.10	2.74	1.35	2.03	1.55
Y	1.06	1.07	0.81	2.30	1.19	1.35	1.11	2.40	1.69
W	0.93	1.06	0.50	0.65	1.01	1.55	0.91	2.31	1.61
H	0.78	0.89	0.97	1.10	1.69	0.15	0.87	1.12	1.12
K	0.86	1.29	1.07	0.37	1.27	0.14	0.38	0.25	0.62
R	1.27	1.29	1.19	0.61	1.21	0.20	0.73	0.40	0.78
Q	1.08	1.21	1.40	0.92	0.74	0.17	0.99	0.62	0.80
N	0.74	0.68	1.12	0.48	0.98	0.26	0.91	0.66	0.91
D	0.77	0.64	0.98	0.81	0.78	0.13	0.81	0.43	0.79
E	0.72	0.70	1.15	0.65	0.73	0.10	0.57	0.64	0.82
