# Supplementary figures and images for: Development of a High-Throughput Assay for Identifying Inhibitors of TBK1 and IKKε
Source: PLoS One. 2012 Jul 30;7(7):e41494. doi: 10.1371/journal.pone.0041494 (PMC3408500; doi:10.1371/journal.pone.0041494)

A)

TBK1 inhibition by the LOPAC library

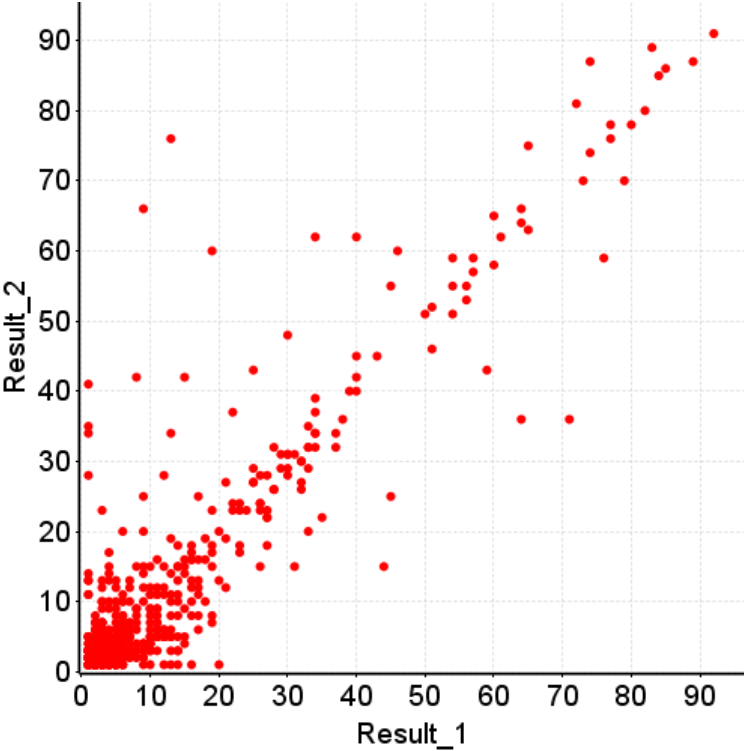

B)

IKKε inhibition by the LOPAC library

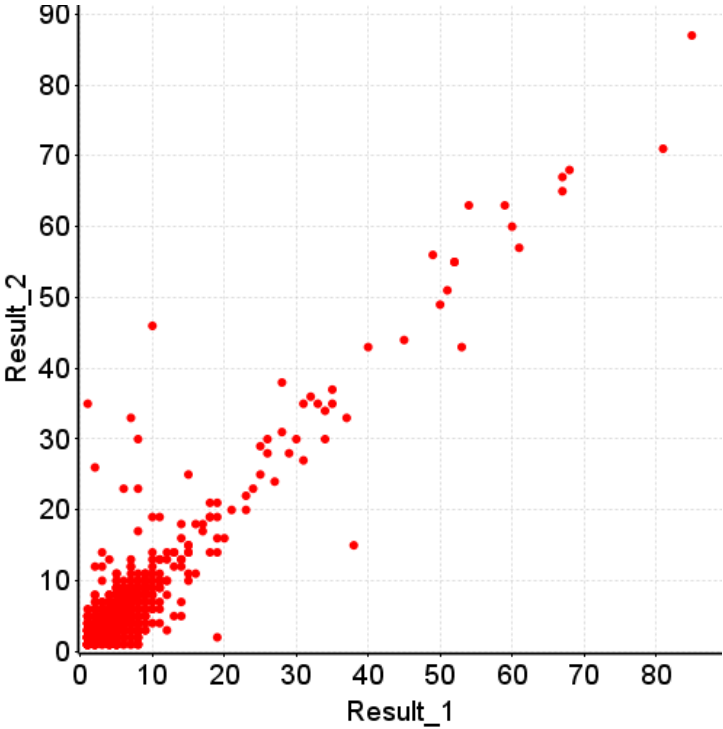

Supplement: Figure S2 — Comparison of duplicate compound assay values. A–B) The LOPAC library was screened in duplicate at 10 µM in a single concentration format against enzymatic reactions of A) TBK1 and B) IKKε. Results from the first determination are shown on the horizontal axis (Result 1) and the second determination is shown on the vertical axis (Result 2). (PDF) [file pone.0041494.s002.pdf]

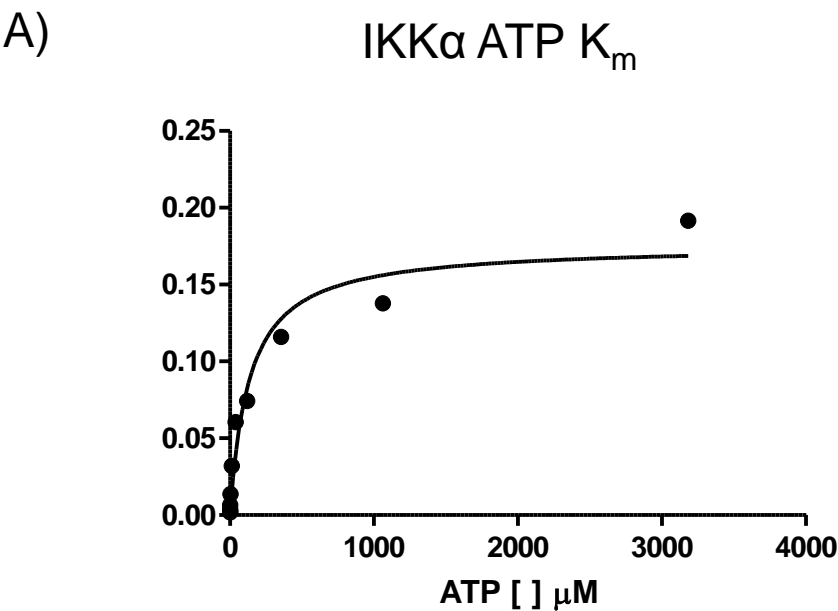

|                  |        |
|------------------|--------|
| Michaelis-Menten |        |
| Best-fit values  |        |
| Vmax             | 0.1758 |
| Km               | 133.7  |

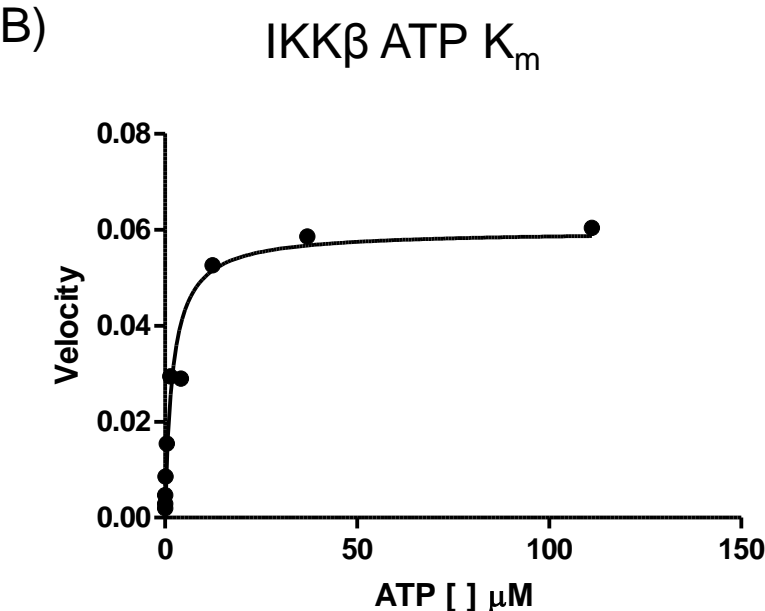

|                  |         |
|------------------|---------|
| Michaelis-Menten |         |
| Best-fit values  |         |
| Vmax             | 0.05972 |
| Km               | 1.981   |

Supplement: Figure S3 — ATP Km determination for IKKα and IKKβ. Enzymatic reactions of A) IKKα and B) IKKβ were incubated at room temperature with 10 ATP concentrations varying from 333 µM to 0.017 µM in three fold dilutions. Reactions were sampled on the Caliper EZReader system at 9.35 minute intervals over a 3 hour period. Percent conversions were calculated from relative heights of product and substrate peaks and used to calculate velocity and ATP Km in Graph Pad Prism. (PDF) [file pone.0041494.s003.pdf]
